# Digital monitoring in rheumatoid arthritis: remote assessment, wearables, and data-driven disease management

**DOI:** 10.1097/BOR.0000000000001166

**Published:** 2026-04-30

**Authors:** Asimina Karampela, Elena Nikiphorou

**Affiliations:** a Barts and the London School of Medicine and Dentistry, Queen Mary, University of London; b Centre for Rheumatic Diseases, School of Immunology & Microbial Sciences, Faculty of Life Sciences and Medicine, King's College London; c Rheumatology Department, King's College Hospital NHS Foundation Trust; d Centre for Education, Faculty of Life Sciences and Medicine, King's College London, London, UK

**Keywords:** digital health, patient-reported outcomes, rheumatoid arthritis, telemedicine, wearable devices

## Abstract

**Purpose of review:**

This review examines the emerging role of digital monitoring technologies in rheumatoid arthritis (RA) management, including electronic patient-reported outcome measures (ePROMs), wearable sensors, and data-driven approaches. The aim is to evaluate how these tools support continuous disease monitoring, timely flare detection, and patient self-management, aligning with treat-to-target principles.

**Recent findings:**

Recent evidence suggests increasing digital health monitoring feasibility through the use of smartphone applications, wearables, and analysis of the multisource data collection. However, barriers such as digital literacy from both patients and healthcare professionals, accuracy of the data collected, correct interpretation still need to be addressed. While digital health has overall shown benefits in terms of appointment distribution, waiting time reduction, continuous monitoring of disease activity, and psychological outcomes, the safe shift to digital monitoring without compromising safety and individuals personal preferences should remain a priority

**Summary:**

Digital monitoring technologies represent a novel shift in RA management, offering continuous assessment beyond traditional clinic visits. While patient acceptance is generally high, implementation challenges include clinician awareness, software integration, and ensuring equitable access. Future directions involve refining predictive algorithms, establishing standardized digital biomarkers, and integrating multimodal data streams for personalized disease management

## INTRODUCTION

Rheumatoid arthritis (RA) is a chronic autoimmune disease, carrying a high disease burden, and often characterized by unpredictable disease course [1]. In current practice, the predominant method of assessment remains in-hospital outpatient clinic visits, with a usual frequency of a couple of months [2], likely limiting the opportunity of closer disease monitoring. Thus, the emerging role of digital monitoring has come to surface, aligning with the treat-to- target principles proposed by EULAR guidelines [3^▪▪^]. Digital monitoring encompasses smartphone applications, wearable sensors, electronic patient-reported outcome measures (ePROMs), teleconsultation platforms, and data analysis of collected metrics. These promising tools aim to offer continuous management, including timely detection of disease flares, while simultaneously promoting self-management. In this review we discuss the following topics: remote assessment tools including ePROMs, wearable devices and sensor-based monitoring, and data-driven approaches. 

**Box 1:**
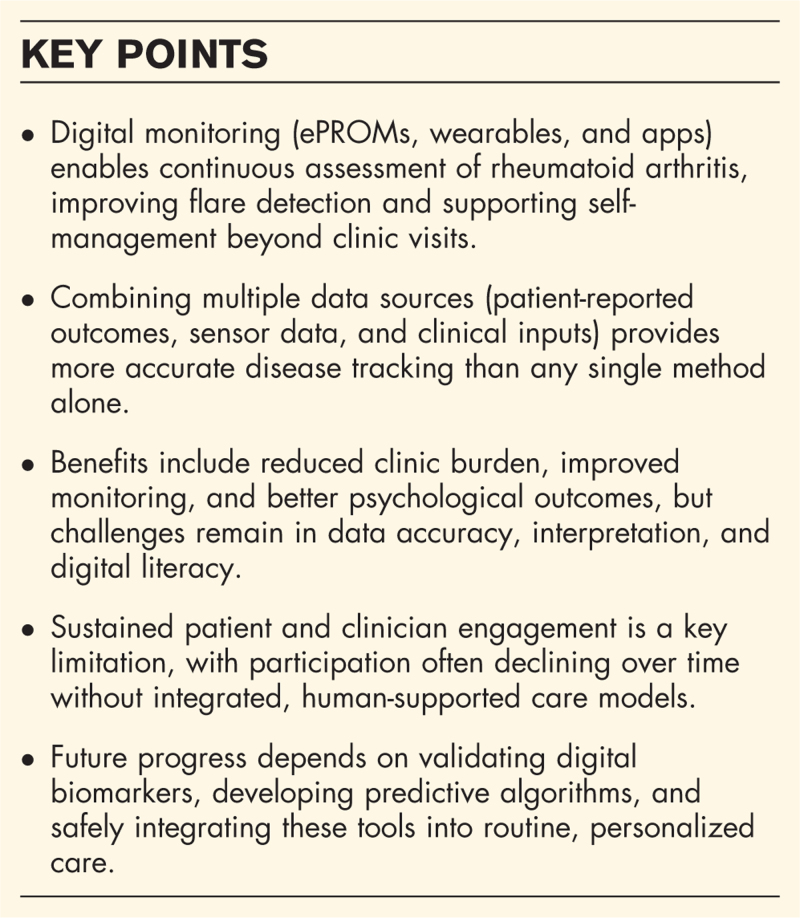
no caption available

## REMOTE ASSESSMENT IN RHEUMATOID ARTHRITIS

Remote assessment in rheumatoid arthritis brings together multiple digital tools that enable continuous monitoring and more responsive, patient-centered care, including ePROMs, use of smartphone applications, and tools effective in measuring disease activity in teleconsultation, while in parallel highlighting the need for multisource assessment for optimal management.

### Electronic patient-reported outcome measures

Patient-reported outcome measures are routinely used to determine the patient's view regarding their experience with their disease. Examples of measurement instruments include Rheumatoid Arthritis Disease Activity Index (RADAI), 36-Item Short Form Survey (SF-36), Routine Assessment of Patient Index Data 3 (RAPID3), and Health Assessment Questionnaire (HAQ) among others [4]. Although historically, PROMs are completed in clinic consultations, new evidence suggests that electronic PROM (e-PROM) use has demonstrated significant positive outcomes in completion over the years, frequency of follow-up appointments and subsequent clinic time and wait time savings in patients with RA [4]. Despite high rates of patient satisfaction with this system, it is still important to not undermine the less than 9% of patients expressing neutrality or dissatisfaction [4]. Interestingly, a greater proportion of clinicians expressed satisfaction compared to the patients; however, both cohorts exhibited a predominantly positive response to their experiences [4]. On the other hand, Krushe *et al.*, revealed barriers in the rheumatologists’ engagement with ePROMs, highlighting unawareness of suitable software solutions, as the main barrier to implementation [5]. Moreover, it has been suggested that clinicians have concerns about disease-related anxiety being increased due to self-reported measures, while high satisfaction with usual management approaches, poor motivation due to lack of symptoms and the system acting as a disease reminder. Nonetheless, both stakeholders found remoting interventions useful; however, it is important to note that evidence of noninferiority of remote health management in terms of RA disease activity and remission rates was conflicting among studies [6^▪▪^].

Of note, PROMs are advised not to be used as a substitute to disease activity as determined by clinical and laboratory measurements [7], while their cumulative use rather than the sole use of one PROM has been found useful [8^▪▪^]. More specifically, a combination of Visual Analogue Scale (VAS) general health/patient global assessment, Health Assessment Questionnaire- Disability Index, quality of life (EQ- 5D) and pain, could be used as a remote screening tool for active RA [8^▪▪^]. However, this combination of multiple dichotomized PROMs resulted in a moderate diagnostic accuracy [area under the receiver operating characteristic curve (AUC-ROC) of 0.76] for distinguishing active from well controlled disease [8^▪▪^]. Further research on this model, identified that the sum of specific PROM items as well as demographic metrics enabled for more accurate discrimination of disease activity [9^▪▪^]. Compared to the model above, this one uses a combination of age, sex, disease duration, visual analogue scale (VAS) general health, 7 Health Assessment Questionnaire-Disability Index items, and VAS pain for the differentiation between well controlled and active disease; An AUC-ROC of 0.89 was shown in the development set, which maintained excellent discriminating ability (AUC-ROC 0.82) in the external validation set [9^▪▪^]. These findings align with previous data which show that wearable-integrated and app-based PROM collection has shown promise in capturing disease fluctuations and improving sensitivity to change compared with static clinic-based assessments [10^▪^].

The RAPID-3 also demonstrates a good discriminating ability, albeit based on both patient and physician assessment. This digital disease activity score (DAS) aligns with DAS-defined disease states which are commonly used in daily practice [9^▪▪^]. RAPID-3 was also used to determine treatment outcomes in patients initiating upadacitinib [11]. Similarly, a case-series evaluated the possibility of using electronic patient-generated pain scores to monitor response to corticosteroid treatment [12]. Therefore, these initiatives hold promising results both in aiding the stratification of the patients to be seen in clinic, but also in treatment monitoring. Nonetheless, this should not override patient and clinical concerns.

Regarding the long-term sustainability of ePROMs in the real-word, the engagement with ePROMs was evaluated in patients with inflammatory arthritis, including RA, through the use of an application. Patient engagement decreased overtime with half of the patients having dropped out by 14.8 months [13^▪^]. The rate of the app downloads was markedly increased during the COVID-19 pandemic, highlighting the need to assess the influence of environmental circumstances in digital health. In contrast, another feasibility study introducing the IMIDoc platform encompasses a broader, more integrated digital intervention, including monitoring with medication management, educational content, bidirectional communication between patients and clinicians, in addition to ePROMs [14]. While this study demonstrated good usability and acceptability, it has not yet detected long-term engagement in routine care. Collectively, these findings highlight a key gap between platform capability and sustained participation in clinical practice.

### Teleconsultation and virtual triage

While teleconsultation compared to face-to-face consultations can entail challenges, it can be a viable and approach for patients, provided there is a safe framework. The RAID score was shown to be strongly correlated with DAS28-CRP score, while a score of <2 reflected good disease control in a teleconsultation setting [15^▪^]. However, this study did not evaluate the triggers to elicit a face-to-face visit, and as mentioned above these scoring systems should always be used along with clinical judgement. As recognized above, the issue of engagement emerges. A mixed methods study evaluated the uptake and views of an online RAID questionnaire, revealing that despite optimal patient engagement, this was not true for the healthcare providers [6^▪▪^], underpinning that efficiency of the digital approach necessitates mutual involvement.

With regards to psychological support for patients with RA, digital cognitive behavioral therapy (CBT) in the form of an application, demonstrated sustained improvements in SF-36 mental (MCS) scores compared to active controls, together with improvements in depression, anxiety, fatigue and social functioning. However, there were no changes in the physical component of SF-36, pain, and disability scores [16]. Overall, this study provides reassurance regarding the safeguarding of the mental health of patients, even from afar.

Interestingly, another feasibility study assessed the significance of digital health measures in patients at-risk of RA [17]. A multimodal digital self-monitoring program was trialed, including a general RA education video, a joint self-examination video, and REMOTRA, a monitoring decision support-system. None of the patients with REMOTRA scores <10 developed RA, yielding a negative predictive value and sensitivity of 100%. However, this should be further refined to ensure diagnostic accuracy is not compromised. Overall, the program was well received, however again engagement declined overtime [17].

Focusing on the reinforcement of self-monitoring via digital methods, the On-demand Program to EmpoweR Active Self-management (OPERAS) app, coupled with a Fitbit^,^ and physiotherapist counselling sessions, demonstrated significantly improved Patient Activation Measure (PAM-13) scores [18], sustained after 27 weeks. This reflects the benefits of multifaceted care, as well as the crucial beneficial role of physiotherapists in remote care. On the contrary, another randomized controlled trial, using an application alone, showed no improvement of Arthritis Self-Efficacy Scale (ASES) scores compared to controls [19^▪^]. This further stresses the importance of the human factor, and the benefits of blended care, as opposed to use of an application alone. Of note, this study reported no increased in pain catastrophizing as a measure to alleviate concerns about the psychological impact of digital monitoring [19^▪^].

## WEARABLES

The use of wearable devices now gives the opportunity for continuous data gathering and utilization. Sharma *et al.* identified patterns in heart rate collected from the wearables, with the capability of distinguishing periods of symptomatic and inflammatory flares, enabling the identification of physiological changes before the onset of clinical disease. However, one of the limitations of this study was the approximate definition of an inflammatory flare as ±7 days of each CRP measurement as part of routine care, underpinning the need for more frequent, scheduled biomarker measurement collection [20]. As alluded to above, the question of adherence to the device use remains, with comfort and digital literacy being potential contributing factors [20]. The need of a ‘trigger” system now comes into question, to determine at which point should each patient need a more thorough review [20]. A feasibility study for wearable accelerometery, demonstrated that this device for use in RA is feasible, has near-perfect adherence by the patients, with reported minimal impact on their daily life, and high willingness for long-term use [21]. Wearables therefore seem to have a practical advantage over the use of a smartphone application. However, this is something to be assessed on an individual basis. Moreover, Creagh *et al.* [22^▪^] expanded on the utility of wearables, by investigating physical activity (PA) metrics of daily-life, using machine learning to characterize symptoms of disease in people with RA compared to healthy controls.

Following the multisource approach, this study proposed that detection of RA severity levels could be improved by augmenting standard patient reported outcomes with sensor-based features as opposed to using PRO assessments alone [22^▪^]. Stradford *et al.* agree that PROMs, wearable data, or clinical measures in isolation are incomplete. In this study they used Fitbit devices to continuously monitor steps, heart rate, sleep patterns, and activity intensity in 150 patients with RA over a 12-week period [23]. The devices demonstrated excellent wear compliance (>90% of days) and successfully captured meaningful variations in activity patterns associated with disease activity changes [23]. The accessibility and affordability of consumer wearables make them attractive for large-scale implementation, though questions remain regarding measurement accuracy and clinical validation.

## DATA-DRIVEN DISEASE MANAGEMENT

With the development of all the aforementioned new technologies used for efficient and monitoring, the transition of this data to meaningful management strategies is imperative. As mentioned above, combining wearable-derived sensor data with patient-reported outcomes has been shown to improve disease classification and severity estimation compared to PROMs alone [22^▪^], which in turn triggers the initiation of management.

Also, the aforementioned emerging digital monitoring systems are extending beyond disease tracking towards prediction and early detection, demonstrating high sensitivity for identifying individuals at risk of developing RA [17]. Although Sharma *et al.* did present that a combination of physiological deviations from baseline may act as early digital trigger signals for disease flares [20], further studies are warranted to establish how these signals are communicated and actioned by the patients and healthcare professionals. Nonetheless, integration into clinical workflows remains a major challenge, with healthcare professionals highlighting concerns regarding data interpretation, validation, and increased workload [24^▪▪^].

## PRINCIPLES OF REGULATION OF REMOTE HEALTH

The development of these new technologies warrant careful frameworks for their implementation into practice, to offer appropriate and safe management. EULAR established points to consider (PtC) for the evaluation and implementation of apps for self-management of rheumatic and musculoskeletal diseases (RMDs) [25^▪▪^]. Key principles, include data protection, the emotional and physical safety of the applications, inclusiveness and consideration of individual user needs, and promotion of communication with healthcare professionals [25^▪▪^]. Expanding on these points, more recent PtC regarding remote care in RMDs overall also highlight some specific points, such as the diagnosis need to the established in a face-to-face visit, as well as the initiation of disease-modifying agents [26].

## CONCLUSION

Digital monitoring in RA is shifting care from intermittent, clinic-based assessment towards a more continuous and data-driven approach. Electronic patient-reported outcomes, wearable devices, and teleconsultation each provide valuable but distinct information, all contributing to identifying changes in disease activity. As shown in this review, these modalities are most effective when used together, rather than in isolation, allowing for a more comprehensive assessment.

An important emerging concept is the move from simple monitoring to actionable disease management. Early evidence suggests that changes in physiological and behavioral data may precede clinical flares, raising the possibility of digital “trigger systems” to prompt timely reassessment. In parallel, predictive models are beginning to support risk stratification and earlier detection of disease. However, there is still a lack of clearly defined thresholds to guide clinical action, and uncertainty remains around how these data should be integrated into routine care.

Sustained patient engagement and clinician involvement are key to the success of digital approaches, as the presence of communication has been shown to improve outcomes,

Overall, digital health technologies offer significant potential to support more personalized and responsive management of RA. Future studies should focus on validating digital signals, defining actionable triggers, and ensuring that increasing data availability translates into meaningful improvements in patient care.

## Acknowledgements

*None.*

### Financial support and sponsorship

*None.*

### Conflicts of interest

*There are no conflicts of interest.*

## REFERENCES AND RECOMMENDED READING

Papers of particular interest, published within the annual period of review, have been highlighted as:

▪ of special interest
▪▪ of outstanding interest
